# Preparation and Properties of Carbon Fiber/Flexible Graphite Composite Grounding Material

**DOI:** 10.3390/ma17194838

**Published:** 2024-09-30

**Authors:** Mian Fan, Huiwen He, Lei Wang, Xianghan Wang, Bo Tan

**Affiliations:** State Key Laboratory of Power Grid Environmental Protection, China Electric Power Research Institute, Hongshan District, Wuhan 430070, China; hehuiwen@epri.sgcc.com.cn (H.H.); wanglei8@epri.sgcc.com.cn (L.W.); wangxianghan@epri.sgcc.com.cn (X.W.); tanbo@epri.sgcc.com.cn (B.T.)

**Keywords:** flexible graphite, carbon fiber, tensile strength, conductivity, corrosion

## Abstract

In this paper, flexible conductive composite materials were prepared from flexible graphite and carbon fiber by mould pressing, and their micromorphology was studied by SEM. The influence of carbon fiber content on the mechanical properties and electrical conductivity of the flexible conductive composite material was studied, and the corrosion rate of the flexible conductive composite material coupling with galvanized steel in soil with different SO_4_^2−^ concentrations was studied. The results showed that the tensile strength reached 5.82 MPa when the mass ratio of carbon fiber to flexible graphite was 1:20, and the volume resistivity achieved 4.76 × 10^−5^ Ω·m when the mass ratio of carbon fiber to flexible graphite was 1:30. With the increase in molding pressure, tensile strength and electrical conductivity had a slight increase. When the flexible conductive composite material was coupled with galvanized steel, sulfate could accelerate the galvanic cell corrosion between the flexible graphite grounding material and galvanized steel. The increase in the sulfate concentration led to more corrosion acceleration. With the increase in corrosion time, the corrosion potential of the flexible graphite grounding material and galvanized steel coupling body decreased to its lowest at 30 days, and then increased gradually. The corrosion current was the highest at 30 days, and then decreased gradually.

## 1. Introduction

In the realm of power system fault occurrences, incidents stemming from lightning strikes on power lines constitute a significant proportion and yield the most severe repercussions [1]. The effective grounding performance of overhead transmission line towers facilitates the efficient dissipation of lightning currents into the ground, thereby furnishing dependable safeguarding for the stable operation of the power system [2,3,4]. In consideration of engineering economics, the current artificial grounding devices for transmission line towers predominantly employ materials such as galvanized steel [5], low-carbon steel [6], or other coated alloys [7,8,9]. However, these metallic materials are susceptible to corrosion and fracture due to the pH value of the soil and the long-term stray currents in the underground transmission corridors, consequently leading to reduced service life and increased maintenance expenditures [10,11].

To address issues such as corrosion susceptibility and uneven current dispersion associated with traditional metallic grounding materials, non-metallic conductive materials (represented by flexible graphite) are receiving increasing attention [12,13,14]. Graphite possesses stable chemical properties, excellent corrosion resistance, conductivity, and impact current tolerance [15]. Additionally, its soft texture allows for close adherence to soil. Huang et al. [16] prepared a new flexible graphite composite grounding material (FGCGM) using high-purity flake graphite with a stranded wire hierarchical structure. Experimental results displayed that this novel non-metallic grounding material holds potential for applications in the field of electrical engineering. Liu et al. [17] developed a graphite cable by combining flexible graphite papers with glass fibers and pressure-sensitive adhesive, and investigated their corrosion behavior in soil when coupled with Zn-coated steel components. Despite these advancements, graphite grounding materials still face practical issues such as poor mechanical properties and susceptibility to detachment under rainwater erosion. In response, researchers have proposed combining carbon fibers; which offer advantages such as high-temperature resistance, abrasion resistance, high specific strength, and high specific modulus; with flexible graphite to fabricate composite grounding electrodes [18,19]. The feasibility of this approach has been demonstrated, although these studies have primarily focused on structural modifications, with less attention given to the impact of material composition on performance.

In this paper, flexible conductive composite materials were prepared from flexible graphite and carbon fiber using mold pressing, and their micromorphology was analyzed using scanning electron microscopy (SEM). The effects of carbon fiber content on the mechanical properties, electrical conductivity, and thermal stability of the flexible conductive composite material were investigated. Additionally, the corrosion rate of the flexible conductive composite material coupling with galvanized steel in soil with varying concentrations of SO_4_^2−^ was studied.

## 2. Experimental Details

### 2.1. Materials

The flexible graphite, designated as model EG-E300, was procured from Qingdao Yanhai Carbon Material Co., Ltd., Qingdao, China. This material is characterized by an 80-mesh screen size and a carbon content that falls within the range of 95–99%. Additionally, it possesses an expansion ratio exceeding 300 mL/g;

The carbon fiber used in this study is T300 grade, designated as SYT45 by Zhongfu Shengying Carbon Fiber Co., Ltd., Lianyungang City, China. The fiber is supplied in the form of a 12 k tows, with a linear density of 800 g/km;

The galvanized steel was procured from Shandong Kangtong Steel Eye Company (Jinan, China).

### 2.2. Preparation of Flexible Graphite Ground Materials

The flexible graphite was expanded at 1000 °C for 15 min in an air atmosphere to form expanded graphite. A certain mass of expanded graphite is mixed with 12 k carbon fiber at three different mass ratios (10:1, 20:1, 30:1), and pressed into graphite paper with thickness of 1 mm by rolling under 5 MPa pressure, then cut into 50 mm × 20 mm × 2 mm sheet reserve.

### 2.3. Preparation of Flexible Graphite Ground Material Coupled with Metal

**Figure 1:**
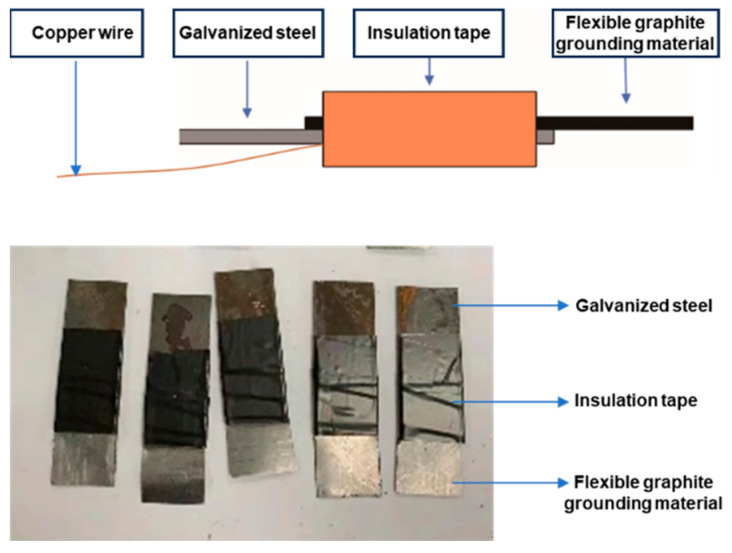
Digital image of the coupled device.

The flexible graphite grounding material was overlapped with galvanized steel sheet of the same size, the contact surface of galvanized steel was welded with copper wire, and the other non-overlapping surfaces were coated with epoxy resin. Bind both ends of the flexible graphite grounding material and galvanized steel with insulation tape to form a coupling body of the flexible graphite grounding material and metal, and label it for later use. The digital image of the coupled device was shown in Figure 1.

### 2.4. Characterization

Scanning electron microscope (SEM): The micromorphology of the composite conductive material was examined using a JSM-5900LV, which is provided by Japan Electronics Co., Ltd. (Takayama, Japan) field emission scanning electron microscope operated at an acceleration voltage of 20 kV and a working distance of 10 mm. The instrument was equipped with both secondary electron (SE) and backscattered electron (BSE) detectors, which allow for the visualization of surface topography and compositional contrast, respectively.

Tensile strength test: A material tensile strength tester (IMT-Tensile 02) which is provided by Dongguan imttest precision instrument Co., Ltd. (Dongguan, China) was used to analyze the tensile strength of the composite conductive material with a testing rate of 2 mm/min.

Volume resistivity test: The circuit shown in the figure is used to test the voltage and current values of cores with different diameters, and the resistivity is calculated by the formula.

$$\rho =\frac{U\pi d^{2}}{4IL}$$

(ρ: volume resistivity; U: voltage; d: conductor diameter; I: current intensity; L: conductor length).

High-temperature resistance test: The carbon fiber reinforced flexible graphite grounding materials were placed in the oven and heated to 100 °C and held at the temperature for 12 h, then the specimens were cooled to 25 °C. The tensile strength test and volume resistivity test were carried out.

Low-temperature resistance test: The carbon fiber reinforced flexible graphite grounding materials were placed in the oven and cooled to −60 °C and held at the temperature for 12 h, then the specimens were heated to 25 °C. The tensile strength test and volume resistivity test were carried out.

Corrosion potential test: Coupling bodies of flexible graphite grounding material and galvanized steel were respectively inserted into simulated soil with SO_4_^2−^ ion concentration of 0.5%, 1%, 1.5%, and 2%. Corrosion tests were carried out for 180 days with 5 samples in each group, during which the observations were made to test the galvanic corrosion potential and corrosion current of coupling bodies at different periods. The morphology of the corrosion surface was observed by SEM. The element content of the corrosion surface was analyzed by X-ray diffraction (XRD) analysis, which was performed by a D8 Advance X-ray diffractometer with Cu-Kα radiation, which is provided by Bruker AXS LTD Germany.

## 3. Results and Discussions

### 3.1. Micromorphology of the Composite Conductive Material

**Figure 2:**
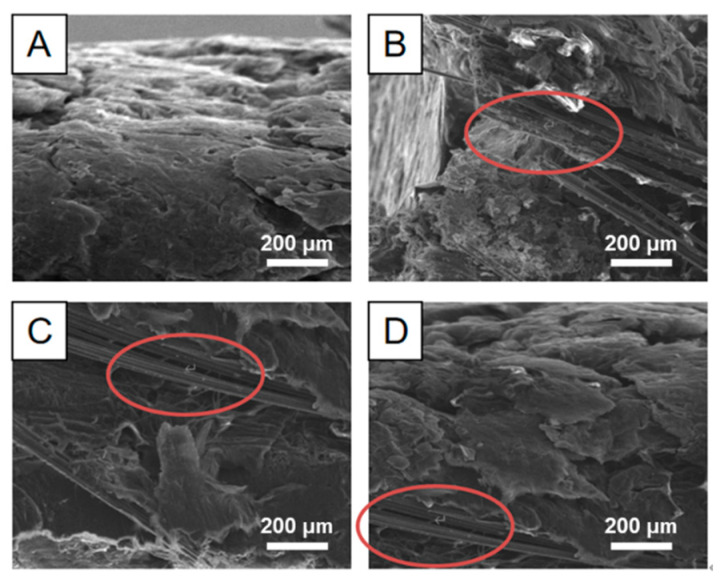
SEM of flexible graphite/carbon fiber combined composite conductive material. (**A**) outside view of composite conductive material; (**B**) tight graphite layer; (**C**) carbon fiber; (**D**) carbon fiber in tight graphite layer.

The micromorphology of the composite conductive material with flexible graphite/carbon fiber is shown in Figure 2. Figure 2A shows the outside view: a tight graphite layer was formed through the mechanical pressing. Figure 2 displays the internal structure of the composite material, where expanded graphite particles, each measuring 200 × 300 μm, could be seen enveloping carbon fiber material. The carbon fibers, indicated by the red ellipsoids in Figure 2B–D, had diameters ranging from 3 to 5 μm. This configuration resulted in a tightly bound composite structure, creating a continuous conductive network.

### 3.2. Mechanical Properties of the Composite Conductive Material

**Figure 3:**
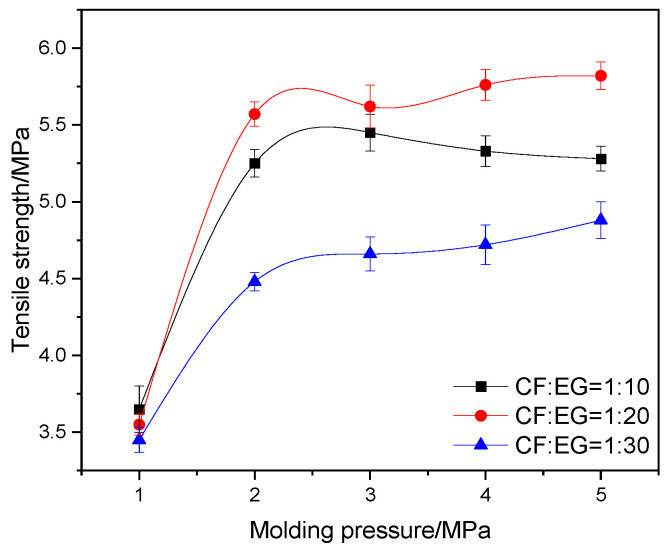
Tensile strength of composite conductive material with different mass ratios of CF/EG prepared by different molding pressures.

To evaluate the mechanical properties of the composite conductive material with flexible graphite/carbon fiber, the composite conductive material combined with different mass ratios of flexible graphite and carbon fiber was prepared with different pressures (1 MPa~5 MPa), and its tensile strength is summarized in Figure 3.

The results demonstrated that an increase in the expanded graphite to composite material ratio from 1:10 to 1:20 led to a slight enhancement in tensile strength for the composite conductive material within a molding pressure range of 2 to 5 MPa. This enhancement could be attributed to the improved wrapping of carbon fibers by the pliable layers of expanded graphite, leading to the formation of a cohesive graphite/carbon fiber interlocking structure that bolsters the overall integrity of the composite. However, upon further increasing the mass ratio of expanded graphite to 1:30, a surplus of graphite remained after the complete wrapping of carbon fibers. This excess graphite, coupled with a reduction in the content of carbon fiber—known for its superior mechanical properties—resulted in a notable decrease in the tensile strength of the fabricated composite.

In addition, when the molding pressure is too low, the continuous dense structure between carbon fiber and graphite is not completely formed, and the structure is loose, resulting in insufficient mechanical properties. When the forming pressure increases, microcracks will form in the structure, leading to stress concentration during the stress process, resulting in the flat line under the molding pressure from 3 MPa~5 MPa. Based on above analysis, the tensile strength of the composite conductive material was found to attain a maximum value of 5.82 MPa under a molding pressure of 2 MPa and with a mass ratio of carbon fiber to expanded graphite (CF/EG) of 1:20.

### 3.3. The Volume Resistivity of the Composite Conductive Material

**Figure 4:**
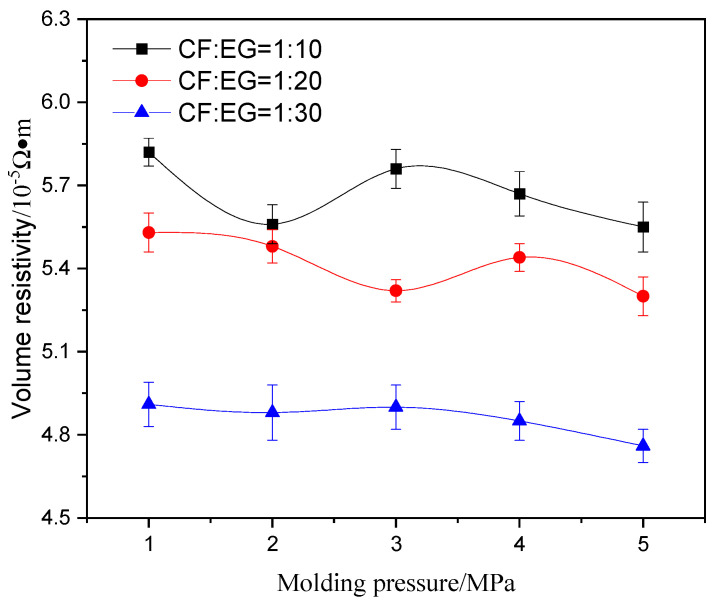
The volume resistivity of composite conductive material with different mass ratios of CF/EG prepared by different molding pressures.

To evaluate the volume resistivity of the composite conductive material with flexible graphite/carbon fiber, the composite conductive material combined with different mass ratios of flexible graphite and carbon fiber was prepared with different pressures (1 MPa~5 MPa), and its resistivity is summarized in Figure 4.

The results indicated that with the increase in the proportion of expanded graphite in the composite material, the volume resistivity of the composite material would decrease; that is due to the conductivity of EG being higher than that of carbon fiber. When the ratio of graphite/carbon fiber was 1:10, the graphite did not completely wrap the carbon fiber, and the conductive network expressed more defects, which led to high volume resistivity; when the mass ratio increased to 1:20, the structure of the conductive network was dense and continuous; when the mass ratio increased to 1:30, more graphite with higher conductivity was introduced, resulting in the composite conductive material with low volume resistivity. Therefore, the volume resistivity of the composite material decreased with the increase in the proportion of expanded graphite.

What is more, an increase in molding pressure led to a reduction in the volume resistivity of the composite conductive material. When the forming pressure was 1 MPa, conductive networks of EG/CF with microcrack defects were formed inside: the internal pores were large, so the volume resistivity was high. When the pressure was 5 MPa, the formed conductive networks were dense and continuous, so the volume resistivity decreased to 4.76 × 10^−5^ Ω·m.

### 3.4. Influence of High- and Low-Temperature Conditions on the Properties of Composite Materials

In general, carbon fiber has a negative expansion coefficient in the axial direction, while graphite has a positive expansion coefficient. In high- and low-temperature environments, due to the different expansion characteristics of the carbon fiber and graphite, internal stress will occur between the two materials at the interface, resulting in microcracks between the layers, resulting in the attenuation of the mechanical properties of the flexible conductor.

**Figure 5:**
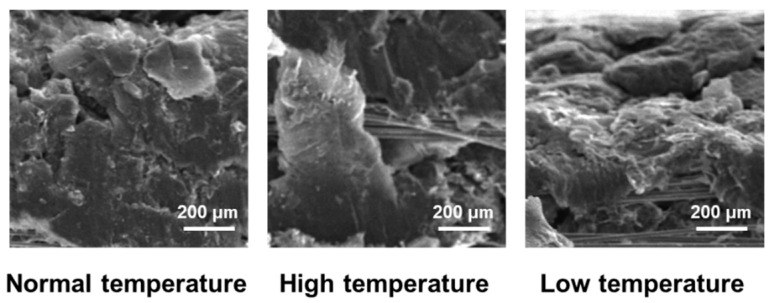
Effect of high- and low-temperature treatments on micromorphology of composite materials.

The micromorphology in Figure 5 indicates that the flexible graphite in the composite material was densely wrapped with carbon fiber before the high- and low-temperature environment treatments, and the composite microstructure was dense and stable. Similarly, after treatment in high- and low-temperature environments, the composite material still presented a flexible graphite-wrapped carbon fiber “sandwich” structure, and there was no overall interface shedding. An explanation is that, during the preparation process, by increasing the pores between the carbon fiber bundles and then pressing and forming flexible graphite on the surface of the yarn body, the flexible graphite can be “penetrated” into the carbon fiber bundle surface and form a mechanical self-locking riveting structure, which can effectively improve the interface bonding force between the graphite layer and the carbon fiber layer in the composite material.

On the other hand, the micromorphology of the composite in Figure 5 shows that the carbon fiber and flexible graphite in the local position still had obvious peeling after high- and low-temperature environmental treatments. This is mainly due to the different expansion characteristics of carbon fiber and graphite; the expansion coefficient of carbon fiber in the axial direction is negative, while the expansion coefficient of graphite is positive. When there was a sudden temperature change, internal stresses would occur between the two materials at the interface, resulting in microcracks between the layers. Therefore, after high- and low-temperature treatments, some carbon fibers had peeled off from the flexible graphite.

The tensile strength and volume resistivity of composites, prepared with a mass ratio of carbon fiber to expanded graphite of 1:20 and molded at a pressure of 4 MPa, before and after high- and low-temperature treatments, are shown in Table 1 and Table 2, respectively. The results indicated that the structural changes in the composite significantly influenced its mechanical and electrical properties. Due to the high- and low-temperature treatments, the porosity of the composite structure increased, so that the tensile strength of the composite after restoring normal temperature decreased slightly, while its resistivity increased to a certain extent.

In addition, the flexible graphite and carbon fiber would have shear force at the interface under high or low temperature conditions, comparatively speaking, because the flexible graphite shrinkage compressed the matrix relatively more tightly during low-temperature treatment; the reduction in mechanical properties and the increase in the resistivity of the composite materials after low-temperature treatment were smaller than those after high-temperature treatment.

### 3.5. Corrosion Mechanism of Composite Conductive Material

Although the flexible graphite grounding material does not itself corrode, it is used in tandem with galvanized steel joints in applications, which constitutes electrochemical corrosion and may cause corrosion of the galvanized steel coupled with it. At the same time, there is more SO_4_^2−^ residue in the flexible graphite grounding material, which will enter the soil with water and increase the electrochemical corrosion rate [20,21,22]. Therefore, the influence of the corrosion rate of flexible graphite grounding material and galvanized steel coupling with the concentration of SO_4_^2−^ was studied.

Table 3 presents the potential and current density of self-corrosion and galvanic corrosion for galvanized steel in a soil test with different SO_4_^2−^ ion contents. Clearly, with the increase in SO_4_^2−^ ion content in the simulated soil medium, both the galvanic and self-corrosion current density of galvanized steel gradually increased. The reason for this result is that the addition of SO_4_^2−^ ions in the simulated soil medium first changes the conductivity of the test soil medium, and, secondly, the sulfate ions in the soil medium will participate in the corrosion reaction of the anode material (reaction rate of catalytic anode metal materials). In addition, the corrosion current density of the flexible graphite grounding material and galvanized steel coupling (20.46~66.81 μA·cm^−2^) was greater than its own corrosion current density (6.70~10.49 μA·cm^−2^). As a result, the corrosion rate of flexible graphite grounding materials and galvanized steel would be accelerated when forming a coupling. In corrosive media, the lower the corrosion potential of the material, the more likely that the material would be corroded. However, the galvanic corrosion potential was higher than the self-corroded potential of the material, but the galvanic current density was higher than the self-corroded current density. The reason is that the galvanic corrosion speed is faster than the self-corroded material because the electrons move faster after the formation of galvanic corrosion. Therefore, the influence of SO_4_^2−^ ions in soil or flexible graphite on galvanic corrosion was much greater than that on the self-corrosion of metal materials.

**Figure 6:**
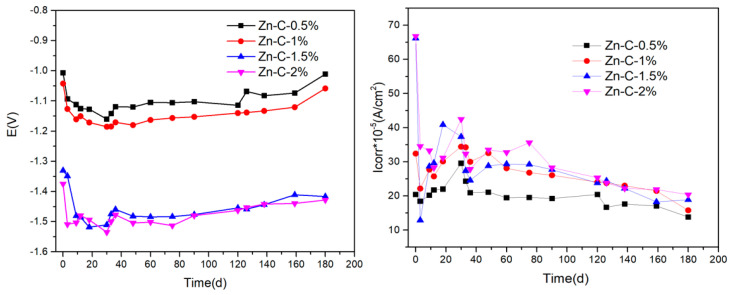
Changes in galvanic corrosion potential and corrosion current between flexible graphite grounding material and galvanized steel over time.

After coupling the flexible graphite grounding material with galvanized steel, the electric couple potential changes in the soil, simulated by SO_4_^2−^ ions with different concentrations, are shown in Figure 6. From Figure 6, when the flexible graphite grounding material and galvanized steel coupling body were tested in the simulated soil, the corrosion potential decreased from −1.01 V to −1.38 V with the increase in SO_4_^2−^ ion concentration in the simulated soil. When the concentration of SO_4_^2−^ ions was 2%, the corrosion potential was the lowest, and the corrosion tendency was greater. With the progress of corrosion time, the corrosion potential decreased to its lowest at about 30 days, which was due to the sulfide in the flexible graphite permeating the soil and resulting in the increase in SO_4_^2−^ ion concentration in the soil. When the corrosion time was further extended, the rate of release and adsorption of sulfide in the flexible graphite grounding material tended to be balanced, and the SO_4_^2−^ ion concentration tended to be stable [23]. These led to the corrosion potential tending to stabilize.

At the same time, the corrosion current of the flexible graphite ground material and galvanized steel coupling body increases with the increase in SO_4_^2−^ ion concentration, indicating that SO_4_^2−^ ions could accelerate the electrochemical corrosion of the coupling body, and the maximum corrosion current appeared at 20–30 days of corrosion. The results indicated that the high concentration of SO_4_^2−^ ions could promote the electrochemical corrosion of the flexible graphite grounding material and galvanized steel coupling.

### 3.6. Morphology Analysis of Corrosion Products

**Figure 7:**
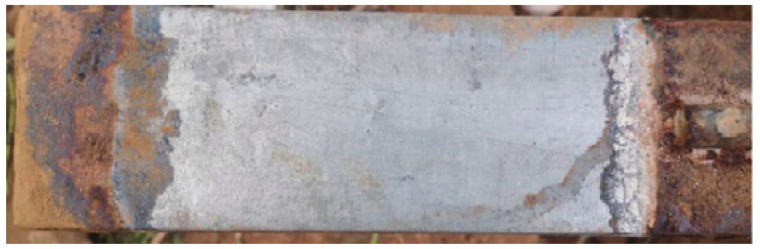
Corrosion morphology of g composite grounding material.

The morphology of corrosion products between the flexible graphite grounding material and galvanized steel are shown in Figure 7. It indicates that severe corrosion occurred at both ends of the galvanized steel with deep corrosion depth, and the overall corrosion position was surface corrosion accompanied by stratification. The bottom layer was black brown with hard texture, and the surface layer was reddish-brown with loose texture. At the same time, it was found that the galvanized steel related to the flexible graphite grounding material; because of the good sealing, the intervention of oxygen was isolated, and there was no corrosion. As the medium permeated from both ends to the middle, the concentration difference was formed, and the corrosion degree decreased gradually.

### 3.7. XRD Analysis of Corrosion Products

**Figure 8:**
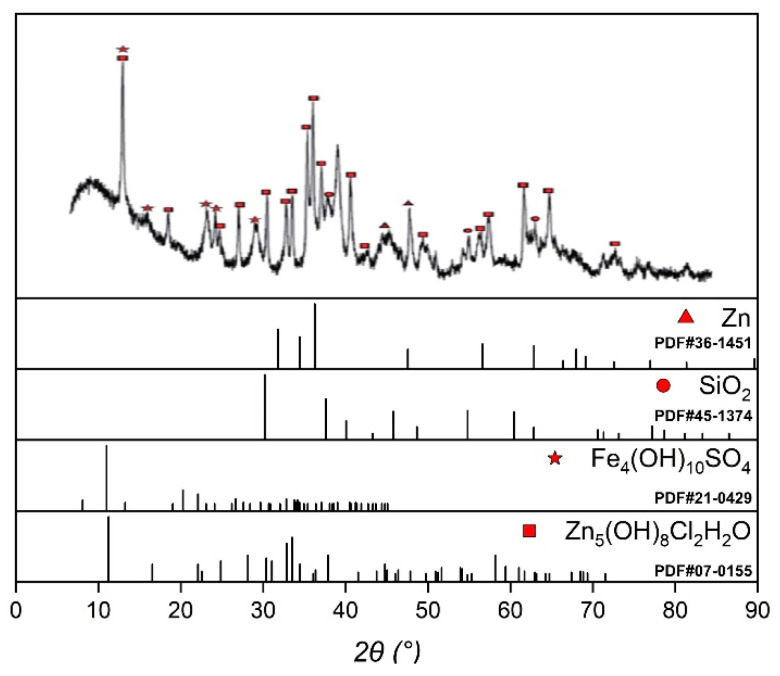
XRD analysis results of composite grounding materials.

The XRD analysis results of the galvanic corrosion products of galvanized steel/flexible graphite grounding material are shown in Figure 8. By consulting relevant literature [5,24,25,26,27,28] and comparing with the standard PDF cards of XRD, it is indicated that the corrosion products were mainly Zn_5_(OH)_8_Cl_2_H_2_O and Fe_4_(OH)_10_SO_4_, among which the outer corrosion products exhibited high oxygen content and relatively loose texture. The presence of Fe_4_(OH)_10_SO_4_ indicated that SO_4_^2−^ ions participated in the corrosion of metal leads, which indicated that the presence of SO_4_^2−^ ions would accelerate the corrosion of metal leads.

## 4. Conclusions

In this paper, flexible conductive composite materials were prepared from flexible graphite and carbon fiber by mold pressing. The results showed that the tensile strength reached 5.82 MPa when the mass ratio of carbon fiber to flexible graphite was 1:20, and the electrical resistivity achieved 4.76 × 10^−5^ Ω·m when the mass ratio of carbon fiber to flexible graphite was 1:30. With the increase in molding pressure, tensile strength and electrical conductivity had a small increase. When the flexible conductive composite material was coupled with galvanized steel, sulfate could accelerate the galvanic cell corrosion between the flexible graphite grounding material and galvanized steel. Therefore, for the sake of reducing the corrosion of flexible graphite grounding materials and metals, on the one hand, the SO_4_^2−^ ion concentration in the soil should be reduced, and on the other hand, the sulfide residues in the flexible graphite grounding materials should be reduced.

## Figures and Tables

**Table 1:** Tensile strength of composite materials before and after high- and low-temperature treatments.

No.	Normal Temperature (MPa)	High-Temperature Treatment (MPa)	Low-Temperature Treatment (MPa)
1	5.5	5.0	5.5
2	5.4	4.9	5.3
3	4.9	4.7	4.6
4	5.4	4.9	5.1
5	5.3	5.0	5.1

**Table 2:** Volume resistivity of composite materials before and after high- and low-temperature treatments.

No.	Normal Temperature (10^−5^ Ω·m)	High-Temperature Treatment (10^−5^ Ω·m)	Low-Temperature Treatment (10^−5^ Ω·m)
1	5.60	6.15	5.94
2	5.67	6.45	6.02
3	5.49	7.28	5.84
4	5.21	6.87	6.15
5	5.12	6.25	5.71

**Table 3:** Self-corrosion and galvanic corrosion data of galvanized steel in soil test with different SO_4_^2−^ ion contents.

Coupling Metal	Potential and Current Density	Concentration of SO_4_^2−^			
		0.5%	1%	1.5%	2%
Galvanized steel	Galvanic potential /V	−1.01	−1.04	−1.33	−1.38
	Couple current density /(μA·cm^−2^)	20.46	32.42	66.20	66.81
	Self-etching potential /V	−1.24	−1.34	−1.43	−1.44
	Self-etching current density /(μA·cm^−2^)	6.70	7.40	9.18	10.49

## Data Availability

Data is unavailable due to privacy or ethical restrictions.
